# Early and very early hepatocellular carcinoma: when and how much do staging and choice of treatment really matter? A multi-center study

**DOI:** 10.1186/1471-2407-9-33

**Published:** 2009-01-27

**Authors:** Fabio Farinati, Adriana Sergio, Anna Baldan, Anna Giacomin, Maria Anna Di Nolfo, Paolo Del Poggio, Luisa Benvegnu, Gianludovico Rapaccini, Marco Zoli, Franco Borzio, Edoardo G Giannini, Eugenio Caturelli, Franco Trevisani

**Affiliations:** 1 Dipartimento di Scienze Chirurgiche e Gastroenterologiche, Università degli Studi di Padova, Padova, Italia; 2 Divisione di Medicina, Ospedale Bolognini, Seriate (BG), Italia; 3 Divisione di Medicina, Ospedale Treviglio-Caravaggio, Treviglio (BG), Italia; 4 Dipartimento di Medicina Clinica e Sperimentale, Università degli Studi di Padova, Padova, Italia; 5 Cattedra di Medicina Interna II, Università Cattolica di Roma, Roma, Italia; 6 Dipartimento di Medicina Clinica, Cardioangiologia, Epatologia, Alma Mater Studiorum-Università di Bologna, Bologna, Italia; 7 Dipartimento di Medicina, Unità di Gastroenterologia, Ospedale Fatebenefratelli, Milano, Italia; 8 Cattedra di Gastroenterologia, Dipartimento di Medicina Interna, Università di Genova, Genova, Italia; 9 Unità di Gastroenterologia, Ospedale Belcolle, Viterbo, Italia; 10 Istituto Oncologico Veneto, Padova, Italia

## Abstract

**Background:**

A consensus on the most reliable staging system for hepatocellular carcinoma (HCC) is still lacking but the most used is a revised Barcelona Clinic Liver Cancer (BCLC) system, adopted by the American Association for the Study of Liver Diseases (AASLD). We investigated how many patients are diagnosed in *"very early" *and *"early" *stage, follow the AASLD guidelines for treatment and whether their survival depends on treatment.

**Methods:**

Data were collected in 530 *"very early" *and *"early" *HCC patients recruited by a multicentric Italian collaborative group (ITA.LI.CA). The Kaplan-Meier method was used to estimate overall survival and the log rank to test the statistical significance of difference between groups. Cox's multivariate stepwise regression analysis was used to pinpoint independent prognostic factors and the adjusted relative risks (hazard ratios) were calculated as well. A statistical analysis based on the chi-square test was used to identify significant differences in clinical or pathological features between patients. A *P*-value < 0.05 was considered statistically significant.

**Results:**

*"Very early" *HCC were 3%; Cox multivariate analysis did not identify variables independently associated with survival. The patients following AASLD recommendations (20%) did not show longer survival. In *"early" *HCC patients (25%), treatment significantly modulated survival (p = 0.0001); the 28% patients treated according to the AASLD criteria survived longer (p = 0,004). The Cox analysis however identified only age, gender, number of lesions and Child class as independent predictors of survival.

**Conclusion:**

patients with *very early" *HCC were very few in this analysis. In most instances they were not treated with the treatment suggested as the most appropriate by the AASLD guidelines and the type of treatment had no impact on survival, even though the number of patients was relatively low and part of the patients were diagnosed before the introduction of the guidelines: this analysis, therefore, might not be considered as conclusive and should be validated. The *"early" *stage group involved more patients, rarely treated according to the guidelines, both overall and also in those diagnosed after their publication; the survival was in part predicted by the type of treatment, with better results in those treated according to AASLD indications.

## Backgrounds

The last 10 years have seen a proliferation of attempts to provide HCC patients with reliable staging and prognostic systems to overcome the inefficiency of the classical Child-Pugh [1], Okuda [2] and tumor-node-metastasis (TNM) [3] staging systems. The most reliable and widely adopted methods for staging HCC are currently the Cancer of the Liver Italian Program (CLIP) [4] and BCLC [5] systems in Europe and the Japan Integrated Staging score (JIS) in Japan [6]. They have been internally and externally validated, both retrospectively and prospectively, and their efficiency has been tested in several clinical and therapeutic scenarios [6-15]. The BCLC system has attracted particular attention because it provides not only a reliable system for staging HCC patients, but also a validated algorithm for the choice of treatment. In the revised version of the BCLC system, released by the AASLD [13], patients diagnosed at the best stages are defined as follows:

- *"very early" *when single node HCC, smaller than 2 cm, in Child-Pugh A class, with no symptoms and lack of change in performance status;

- *"early" *when single node HCC, smaller than 5 cm, or up to 3 nodes < 3 cm each, in Child-Pugh A-B class, with no symptoms and lack of change in performance status.

In the AASLD guidelines, patients with *"very early" *disease are candidates for resection, unless they have portal hypertension and/or increased bilirubin levels, in which case either liver transplantation or locoregional percutaneous treatments are recommended, depending on their age and associated diseases. Patients with *"early" *disease, when presenting portal hypertension or increased bilirubin that advise against the surgical option, should be given locoregional treatment or transplant as well.

The aims of this retrospective multi-center study were to ascertain:

- the proportions of HCC patients presenting with *"early" *and *"very early" *HCC in the cumulative experience of ten Italian institutions, three of them acting as primary referral centers and seven as both primary referral and third-level centers;

- how was the management in relation to the AASLD (or previous BCLC) treatment guidelines;

- whether the choice of treatment really has a crucial impact on the survival of patients in these two stages, which is the most effective treatment and whether adherence to the AASLD guidelines has an impact on survival.

## Methods

This study retrospectively analyzed data collected prospectively concerning 1834 HCC patients (482 females, 1352 males) recruited from January 1986 to December 2004 at 10 clinical institutions forming the ITA.LI.CA (Italian Liver Cancer) group. The *diagnosis *of HCC was histologically confirmed in 939 cases. In the remainder, it was based on the guidelines on HCC management, and obtained by at least two imaging techniques with typical features for HCC or combining a diagnostic increase in alfa-fetoprotein (AFP) (> 200 ng/mL) with typical features detected by one imaging technique [14]. The *modality of cancer diagnosis *was defined as "surveillance" when HCC was detected during routine follow-up (6-monthly or yearly clinical examination and imaging), "incidental" when an asymptomatic neoplasm was discovered outside a surveillance program, and "symptomatic" when HCC was diagnosed because of the onset of symptoms.

*Tumor size *and *number of lesions *were determined on the basis of the imaging procedures performed, considering the diameter of the largest lesion in cases of multiple nodules. *Tumor stage *was determined according to the tumor-node-metastasis (TNM) [3], and Cancer of the Liver Italian Program (CLIP) [4] staging systems, as in previous ITA.LI.CA reports [16,17]. *Tumor stage *was retrospectively correlated with the BCLC system [5], given the availability of reliable data on patients' symptoms and performance status in the original data base. We considered portal hypertension (as clinically suggested by the presence of esophageal varices) or bilirubine level < 2 mg/dl as exclusion criteria for resection.

*Virological status *regarding hepatitis B virus (HBV) and hepatitis C virus (HCV) infection was determined on the basis of routine virological tests. *Alcohol abuse *was considered as the long-standing consumption of more than 80 g ETOH/day in males and 40 g in females. The *Child-Pugh class*, the presence of relevant *symptoms *(ascites, jaundice), "constitutional syndrome" (fever, weight loss and pain) or of *portal thrombosis*, *extra-hepatic metastases*, or *associated nonhepatic disease*, were recorded.

*First treatment *after diagnosis, i.e. transplantation (OLTx), surgical RESECTION, radiofrequency-mediated thermal ablation (RFTA), percutaneous ethanol injection (PEI), transcatheter arterial chemoembolization (TACE), combinations of different treatments and best supportive care (BSC) were recorded together with the *survival *in months from the time of diagnosis. Treatments for HCC were chosen either in accordance to the guidelines or taking into account specific neoplastic, clinical and biochemical features of each patient. This being a partly retrospective and partly prospective observational study no attempt was done to standardize the treatment choice in each center.

The Kaplan-Meier method was used to estimate overall survival (median and 95% CI), defined as the interval between HCC diagnosis and death or last follow-up visit. To test the statistical significance of differences in survival between groups, the log rank method was utilized. The variables considered in the Cox's multivariate stepwise regression analysis to pinpoint independent prognostic factors were:

- age;

- gender;

- etiology;

- tumor size (diameter of largest nodule, scored as: 1 = < 2 cm, 2 = 2–3 cm, 3 = > 3 cm);

- number of lesions (1, 2–3);

- Child-Pugh class (A/B);

- CLIP score (0–3);

- type of treatment (OLTx, RESECTION, RFTA, PEI, TACE, best supportive care [BSC], others).

An internal validation analysis was also performed in a set of 100 consecutive "*early" *HCC patients diagnosed in an more recent time period (included from January 2002 and December 2004). This was not done on patients with *"very early" *HCC due to the small number of patients available for the analysis.

The adjusted relative risks (hazard ratios) and their 95% confidence interval (CI) were calculated for variables independently correlated with survival. A statistical analysis based on the chi-square test was used to identify significant differences in clinical or pathological features between differently treated patients. A *P*-value < 0.05 was considered statistically significant.

The software utilized was SPSS 14.0 for Windows.

The ITA.LI.CA database management conformed to the past and current Italian legislation on the privacy and the study conformed to the ethical guidelines of the Declaration of Helsinki. The study was approved by the ethic committee of the participating Institutions.

## Results

Based on the above-described AASLD criteria, two groups of patients were singled out, one with *"very early" *HCC, including 65 patients (3% of the whole series), and the other with *"early" *HCC, encompassing 465 patients (25%). Therefore, these patients accounted overall for the 28% of all cases diagnosed at the units participating in the study.

The features of the two subgroups are given in Tables 1.

**Table 1:** Distribution of "*very early" *and "*early" *HCC, according to the different variables.

		*Very Early *HCC (65)		*Early *HCC (465)	
Variables		N	%	N	%
**Gender**	Male (M)	43	66	316	68
	
	Female (F)	22	34	149	32

**Age**	< 60	16	24	107	23
	
	60 – 70	31	48	214	46
	
	> 70	18	28	144	31

**Disease Aetiology**	HBV	4	6	42	9
	
	HCV	44	68	274	59
	
	Alcohol abuse	7	11	51	11
	
	Alcohol and virus	4	6	56	12
	
	Mixed and others	6	9	42	9

**HCC diagnosis**	Incidental	6	9	116	25
	
	Surveillance	58	89	326	70
	
	Symptomatic	1*	2	23*	5

**Alfafetoprotein**	< 20 ng/ml	31	47	228	49
	
	20–200 ng/ml	27	42	205	44
	
	> 200 ng/ml	7	11	32	7

**Bilirubin**	< 2 mg/dl	62	95	398	85
	
	≥2 mg/dl	3	5	67	15

Lesion number	1	65	100	344	74
	
	2–3	0	0	121	26

**Tumor size**	≤2 cm	65	100	158	34
	
	2–3 cm	0	0	202	43
	
	> 3 cm	0	0	105	23

**Child-Pugh class**	A	65	100	331	71
	
	B	0	0	134	29

**CLIP score**	0	61	93	243	52
	
	1	4	7	167	36
	
	2	0	0	47	10
	
	3	0	0	8	2

**Type of treatment**	OLTx	3	5	11	2
	
	RESECTION	8	12	75	16
	
	RFTA	14	22	58	12
	
	PEI	20	30	106	23
	
	TACE	17	26	128	28
	
	PEI + TACE	-	-	25	6
	
	BSC	3	5	62	13

OLTx: orthotopic liver transplantation; RFTA: radiofrequency-mediated thermal ablation;

PEI: percutaneous ethanol injection; TACE: transcatheter arterial chemoembolization; BSC: best supportive care.

Taken together, *"very early" *and *"early" *HCC patients showed the following characteristics:

- a median overall survival of 46 months (95% CI 42–50);

- patients with nodes < 2 cm survived significantly longer (median 65 months, 95% CI 49–80) than those with nodes 2 to 3 cm (43 months, 95% CI 38–48) or larger than 3 cm (46 months, 95% CI 41–50, p < 0.001);

- Child A patients had a significantly longer survival (median 50 months, 95% CI 44–54) than Child B subjects (26 months, 95% CI 21–31, p < 0.0001), irrespective of tumor size.

### "*Very early*" disease

**Figure 1:**
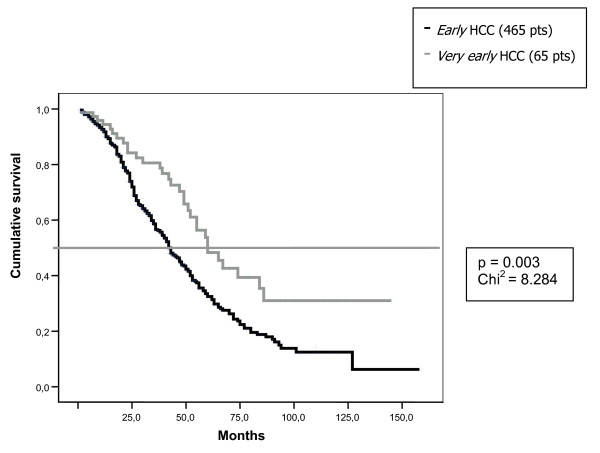
**Survival rate in "*very early*" and "*early*" HCC**. Patients with *"very early" *HCC survived longer (median 60 months, 95% CI 47–72) than patients with "*early*" disease (42 months, 95% CI 38–46). The difference is highly statistically significant (p < 0.003).

*"Very early" *HCCs were more frequently diagnosed in patients under surveillance (p = 0.002) and survived longer (median 60 months, 95% CI 47–72) than patients with "*early" *disease (42 months, 95% CI 38–46)(p < 0.003) (Fig. 1). The percentage of *"Very early" *HCCs did not increase over time and it was stable at 3% also in the 2002–2004 period, chosen to evaluate any trend.

**Figure 2:**
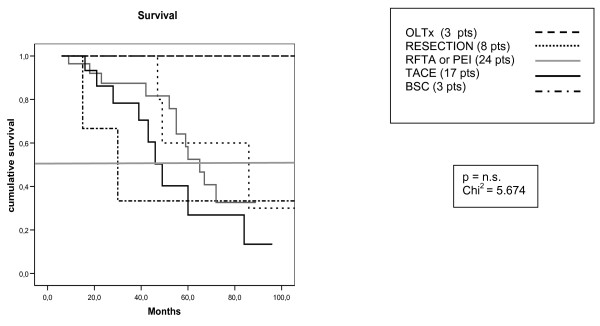
**Survival curves in patients with *very early *HCC treated with different modalities**. The difference in survival according to treatment is not statistically significant (p = 0.06) [OLTx median survival 90 months; resection 86 months (CI 16–156), RFTA and PEI 65 months (CI 54–76), TACE 60 months (CI 37–83), BSC 15 months (CI 6–43)]. (OLTx: orthotopic liver transplantation; RFTA: radiofrequency-mediated thermal ablation; PEI: percutaneous ethanol injection; TACE: transcatheter arterial chemoembolization; BSC: best supportive care).

Among *"very early" *HCC patients, there was a borderline (p = 0.06) difference in survival between patients receiving OLTx, RESECTION, PEI, RFTA, TACE and BSC (Fig. 2). OLTx gave the best results, with a median survival of 90 months; resection followed with 86 months survival (CI 16–156). In this group the survival curves of RFTA and PEI were considered together, given the small number of patients for each treatment: they obtained a median survival of 65 months (CI 54–76). Patients undergoing TACE had a survival of 60 months (CI 37–83). Only 3 patients were assigned to BSC, due to old age (> 80 years) (1 pt), multiple associated pathologies (1 pt) or unknown reasons (1 pt). their median survival was 15 months (CI 6–43).

However, when the 3 cases treated with OLTx and the 3 cases who underwent BSC are disregarded, then the borderline difference disappears (p = 0.858). Cox's multivariate analysis showed that no variable was independently associated with survival.

The group of patients treated according to the AASLD recommendations (20%) did not survive longer than that of patients treated differently (80%)(the subgrouping is shown in Table 2).

**Table 2:** Number of "*very early" *HCC patients treated in adherence with the AASLD indications.

		ACTUAL TREATMENT				
AASLD INDICATIONS		RESECTION	OLTx	PEI/RFTA	TACE	BSC
	
	RESECTION	***5 (13%)***	1(2%)	24(59%)	10(24%)	1(2%)
	
	OLTx	2(33%)	***2(33%)***	-	2(33%)	-
	
	PEI or RFTA	1(7%)	-	***6(46%)***	4(31%)	2(16%)

OLTx: orthotopic liver transplantation; RFTA: radiofrequency-mediated thermal ablation;

PEI: percutaneous ethanol injection; TACE: transcatheter arterial chemoembolization; BSC: best supportive care

AASLD indications for treatment are reported in rows; percentages of patients treated per each modality are calculated on the totality of patients having that specific indication.

The adherence to AASLD indication was evaluated when all data were available (60/65 pts – 91%).

### "*Early*" disease

**Figure 3:**
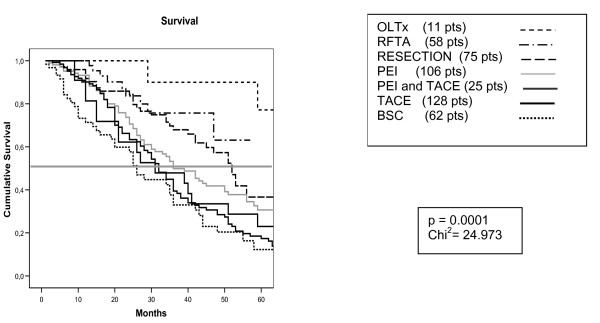
**Survival curves in patients with *early *HCC**. The difference in survival among groups according to treatment modality is highly statistically significant (p = 0.0001) [OLTx mean survival 106 months (CI 89–124); Surgical resection median survival 52 months (CI 45–58), RFTA 62 months, PEI 44 months (CI 37–50), TACE alone 34 months (CI 29–39), TACE+PEI 41 months (CI 27–54), BSC 28 months (CI 17–39)]. (OLTx: orthotopic liver transplantation; RFTA: radiofrequency-mediated thermal ablation; PEI: percutaneous ethanol injection; TACE: transcatheter arterial chemoembolization; BSC: best supportive care).

In cases of *"early" *HCC, a significant difference in survival emerged among patients treated with the different therapies (p = 0.0001, Fig. 3), OLTx patients achieving the best outcome (mean survival 106 months, 89–124 CI). Surgical resection obtained a median survival of 52 months (CI 45–58), RFTA of 62 months and PEI of 44 months (CI 37–50). Worse results were reached with TACE, either alone (median survival 34 months, CI 29–39) or in combination with PEI (median survival 41 months, CI 27–54). Here again, patients were assigned to BSC due to very advanced age (> 80 years) (5 pts), multiple comorbidities (23 pts) or unknown reasons (34 pts), and they got the worst survival (28 months, CI 17–39).

In an attempt to identify possible causes of the low efficacy of TACE, we found that patients treated with TACE:

- were more often Child B (p = 0.0016): 40% (52/128) versus 20% (54/275) in the other treatment groups;

- had a higher prevalence of large nodules (> 3 cm) (p < 0.005): 19% (23/128) versus 1% (4/275) in patients on other treatments.

We therefore considered the subgroup of patients treated with TACE and presenting with a Child A cirrhosis and small nodules (≤ 3 cm), as compared with a group with similar characteristics but treated with RESECTION, PEI or RFTA, and there was still a significant difference in survival, thus confirming that TACE is the treatment coinciding with the worst survival (p < 0.03). However, when we considered patients treated with TACE and BSC alone, TACE was linked with a significantly longer survival (p = 0.04).

Finally, potential differences in survival were also sought by comparing patients treated with radical therapies, that is RESECTION, RFTA or PEI: the first two treatments offered a similar chance of survival (median 52 months [95% CI 47–57] for RESECTION, median not applicable for RFTA [survival > 50% at 60 months]) and they provided a significantly longer survival than PEI (median 36 months [95% CI 28–44]) (p = 0,012). The patients included in RFTA and RESECTION groups did not revealed significant differences in terms of Child-Pugh class or number and size of HCC lesions.

The Cox's multivariate analysis showed that age, gender, number of lesions and Child-Pugh class were independent predictors of survival (p = 0,001; p = 0,05; p = 0,012 and p = 0,009, respectively), while the type of treatment was not. Age increases the death risk by 2% for each year, male gender by 22%, while lesions number > 1 increases the death risk by 47% and Child-Pugh class by 70%. Then again we subgrouped the patients according to whether their treatment complied with the AASLD algorithm (Table 3). Over one third of the patients (36%) are treated with TACE, outside the AASLD indications, and 13% undergo medical treatment and/or BSC.

**Table 3:** Number of "*early" *HCC patients treated in adherence with the AASLD guidelines.

		ACTUAL TREATMENT				
AASLD INDICATIONS		RESECTION	OLTx	PEI/RFTA	TACE/ PEI+TACE	BSC
	
	RESECTION	***30 (28%)***	1 (1%)	44 (40%)	27 (25%)	7 (6%)
	
	OLTx	6 (9%)	***6 ***(9%)	9 (14%)	34 (52%)	11(16%)
	
	PEI or RFTA	32 (13%)	4 (2%)	***83 (34%)***	88 (36%)	37(15%)

OLTx: orthotopic liver transplantation; RFTA: radiofrequency-mediated thermal ablation; PEI: percutaneous ethanol injection; TACE: transcatheter arterial chemoembolization; BSC: best supportive care.

AASLD indications for treatment are reported in rows; percentages of patients treated per each modality are calculated on the totality of patients having that specific indication.

The adherence to AASLD indication was evaluated when all data were available (419/465 pts – 90%).

### Internal validation analysis

Given the risk that the inclusion of patients diagnosed in such a long time span may lead to a bias due to the different approach to the treatment over time, both in terms of management of HCC (before/after the publication of the guidelines) and in terms of available therapeutic techniques, an internal validation analysis on a series of about 145 patients consecutively diagnosed in a much more recent period was introduced. The set of consecutive patients with "*early*" HCC included in the internal validation did not show any statistically significant difference from the whole series with respect to the baseline characterization (mean age, gender and so on). The statistical evaluation of the series of "*early*" HCC patients confirmed:

- the significant difference in survival in relation to the treatment applied (p = 0.003), with OLTx obtaining the best survival (79 months), resection and RFTA a median of 45 months, and BSC the worst results, with 12 months survival (Table 4);

**Table 4:** Median survival (months) in the 145 patients with "*early" *HCC of the internal validation analysis according to the type of treatment.

Type of treatment	N	Median survival (months)	CI 95%	
**OLTx**	5	79	66.211	91.556

**RESECTION**	17	45	34,327	53,254

**RFTA**	46	45	40.394	51.247

**PEI**	16	37	14.384	59.616

**TACE**	41	44	34.518	53.482

**PEI + TACE**	10	36	32.006	39.994

**BSC**	10	12	9.078	14.922

**Total**	145	45	40.708	49.292

OLTx: orthotopic liver transplantation; RFTA: radiofrequency-mediated thermal ablation; PEI: percutaneous ethanol injection; TACE: transcatheter arterial chemoembolization; BSC: best supportive care.

The difference in survival among treatment groups is statistically significant (p = 0,003). In the COX multivariate analysis however only age and Child-Pugh class (p = 0.025 and p = 0.011, respectively) were selected as independent predictors of survival.

- the lack of any significant difference with respect to the compliance to the AASLD guidelines;

- the role as independent predictors of survival in the COX multivariate analysis of the two variables that were more significantly associated with prognosis in the whole series, i.e. age and Child-Pugh class (p = 0.025 and p = 0.011, respectively), and, conversely, the lack of a significant impact of the choice of treatment.

## Discussion

The main goals of a staging system for a neoplastic disease are: 1) to give patients a clear prognostic information; 2) to enable the comparison of the results of different treatments and 3) to suggest a specific treatment for a given stage of the disease.

Since the literature contains sound evidence of the validity of both the CLIP and the BCLC staging system, now revised in the AASLD guidelines, the claimed superiority of the BCLC over other similarly-validated systems, such as the CLIP, would be mostly based on the last of the above points. Whether or not either of these two systems is clearly superior, supporters of the BCLC maintain indeed that this system is the only one to link the patient's stage with a clear-cut treatment indication.

Generally speaking, the characteristics of patients recruited at a given center have an influence on the efficiency of a given staging system. Different staging systems will be more suitable, depending on where and why they are used [18]. For instance, it is now generally accepted that the CLIP system works better for advanced HCC and the BCLC for early disease undergoing surgery and in non cirrhotics [19,20]. Therefore, some authors go as far as to suggest that it might be useful to establish different staging systems, for patients undergoing resection or other ablative therapies, and for those undergoing palliative treatment [12].

For the BCLC staging system, however, the bottom line is: can we be fully confident that the proposed stage-related treatment indications are so helpful that adhering to them will give patients the best possible chances of survival?

Regarding the revised *"very early" *HCC stage, our results suggest the following:

1. The proportion of patients with *"very early" *HCC is only 3% in a large series collected by primary referral centers involved in recruiting patients with non advanced disease. As this figure would probably decrease at less experienced centers, our information makes rather feeble the epidemiological relevance of this stage in clinical practice. It is doubtful whether the trend toward an earlier diagnosis of HCC observed in centers where surveillance of cirrhotics is performed would significantly modify the overall figure [21], given that this did not happen in our series, at least until 2004.

2. Survival rates in patients with *"very early" *HCC are satisfactory irrespective of the type of treatment. In the univariate analysis, the choice of treatment, overall, shows a borderline impact on survival. However, when patients who underwent OLTx, that should not be indicated in this stage of the disease with very few exceptions, and those who underwent BSC are disregarded, any difference disappears. In the multivariate analyses the choice of treatment has no impact on survival, even when including BSC. However, being low the number of patients with "*very early*" HCC we might have missed the expected difference in survival among treatment groups and prospective validation analysis is needed to define the best treatment in this subgroup of patients.

3. Finally, the percentage of patients treated according to the AASLD recommendations was rather small. Since this result was common to *"early" *HCCs, it will be commented later.

The picture is slightly different for patients with *"early" *disease, who account for a substantial share of HCCs. Here the survival was highest after OLTx, therapeutic procedure performed only in 2% of the patients, possibly as a result of reduced organ availability, comorbidity or advanced age. Same considerations apply also to the group of *"very early*" HCC. RESECTION followed in terms of efficacy, then percutaneous treatments, while survival was lowest in patients treated with TACE or BSC. Actually, patients treated with TACE were more likely to have larger tumors and Child B status, with no difference in the number of nodules, but the difference between TACE, RESECTION and percutaneous treatments remained even after correction for these two factors, in patients in Child A disease and small tumors. Nonetheless, TACE offered a prognostic advantage with respect to BSC, suggesting that it still is of some therapeutic value.

Another point much debated in the literature concerns the comparison between RESECTION and locoregional treatments. This point was not addressed directly in our study but, by comparing the results of RESECTION, RFTA and PEI, we found that the first two treatments performed better than PEI. This would be, at least in part, in line with data recently provided by prospective randomized studies, showing that RESECTION was as effective as percutaneous treatments and RFTA superior to PEI [22-25]. Our retrospective observations just confirm what above and it is worth bearing in mind, however, that RESECTION has hitherto been the treatment of choice for patients with limited disease, though these findings insinuate that recommending RESECTION in the current practice as the front-line treatment for patients with *"early" *(and even more for those with *"very early"*) HCC is not so confidently supported by the literature [26].

The above considerations are confirmed by the result of multivariate Cox's analysis, where the type of treatment did not emerge as independent predictor of survival, implying that, in the relatively homogeneous group of patients with *"early*" HCC, its prognostic impact is overcome by that of other more powerful factors, e.g. age, gender, Child-Pugh class and tumor burden.

In a recent work, Wang et al analyzed a large cohort of patients to assess the impact on survival of treatment choice in the different BCLC stages [27]. A clear-cut difference in survival with respect of the therapeutic option was found for *"early" *HCC patients, as well as in our study; for *"very early" *HCC patients the Authors reported a borderline significance for the decreasing linear trend in survival, with a distinct difference between surgery and TACE, but not between surgery and percutaneous treatments. Furthermore, as the Authors state, the analysis presents some limitations, both in the stage assignation of patients and in the choice of treatment (for instance, no patients were transplanted, and very few underwent percutaneous ablation), so that the results can not be considered as conclusive. In any case, also in their experience, the percentage of patients with *"very early" *HCC was negligible (3%), as in our series.

An additional point to make is that the percentage of patients with *"early" *disease treated according to the AASLD guidelines, albeit higher than in *"very early" *HCC, remained relatively small. While for "*very early*" HCC no survival benefit was found for patients treated according to the guidelines, in *"early" *HCC the difference between the two subgroups was statistically significant, but again, without being identified in the multivariate analysis as an independent predictor of survival.

Our study presents some limitations, due to the wide time interval of patients recruitment: it is well-known that in recent years diagnostic and therapeutic techniques have significantly developed, leading to a wider identification of early HCC stages and allowing more frequently the application of radical treatments. Furthermore, our data collection began before the publication of BCLC staging system, and this may have influenced our results.

Being aware of this and in order to minimize any bias, an internal validation analysis was carried out in a series of 100 consecutive *"early" *HCC patients, diagnosed in a more recent period: even in this case the principal data obtained in the whole patients group were basically confirmed.

Why do so few centres follow the AASLD recommendations? We can suggest three explanations:

1) Our series was recruited before the emanation of AASLD guidelines and, although its therapeutic algorithm had been proposed before by the Barcelona group, it was still under debate and revision. Indeed, most ITA.LI.CA clinicians followed the Italian guidelines [14] which had been available earlier. We tried to limit this bias selecting a more appropriate series of patients, but further validation analysis on recent years may be useful to understand how much the application of the guidelines is different from our results.

2) The experience matured in clinical practice could have suggested that *"very early" *HCC occurring in well compensated patients can be efficiently treated with different approaches. In line with our result, a recent randomized controlled trial has shown that RESECTION and PEI ensure the same survival to cirrhotic patients with one or two nodules ≤2 cm each [28].

3) Finally, the therapeutic choice concerning *"early" *tumors stems from the day-to-day experience gained by operators who evaluate the position and boundaries of the HCC node(s), the extent of vascularization and portal hypertension, concomitant diseases, the patient's age or will, local expertise and resources.

This may have produced the gap between theoretically ideal and real-life treatment decisions. Even though, generally speaking, guidelines should not be considered as mandatory advices, should we try harder to stick to the AASLD recommendations? Based on the outcome of this study, it is hard to say.

## Conclusion

In short, the data deriving from this analysis of one of the largest available databases on HCC demonstrate that:

- patients with *"very early" *HCC are presently very few, probably too few to make a definition of this stage of the disease clinically useful. As recently suggested however [29], the situation might change since we foresee a larger share of HCC patients diagnosed in this stage as a result of the wide implementation of surveillance programs, at least in the western world. With some caution needed in the evaluation of this set of data, given the relatively small number of patients included, the survival rates in these patients are satisfactory not only with OLTx, resection and RFTA [30], but whatever the treatment, in contrast with some previous reports, so that the stage-treatment link appears to be of little consequence;

- patients with *"early" *disease are a relatively sizable share of all HCC patients diagnosed. For them using the AASLD guidelines coincides with better survival; as for the types of treatment, these can be placed in order of efficiency, with OLTx at the top and TACE, generally used in patients with more advanced disease, at the bottom. Nevertheless, the treatment type and the adherence to the AASLD therapeutic algorithm appear to be less important in predicting survival than patient's age, gender, number of nodes (i.e. tumor burden) and Child-Pugh class (i.e. liver function).

## Competing interests

The authors declare that they have no competing interests.

## Authors' contributions

FF, AS, AB, AG planned and carried out the study, performed the statistical analysis and wrote the manuscript; FT contributed in drafting the manuscript and revising it; MADN, PDP, LB, GR, MZ, FB, EG, EC read the manuscript and made appropriate suggestion. All Authors recruited the patients and participated to collection of data. All Authors have given final approval of the version to be published.

## Pre-publication history

The pre-publication history for this paper can be accessed here:

http://www.biomedcentral.com/1471-2407/9/33/prepub
